# New York State

**Published:** 1896-08

**Authors:** 


THE NEW YORK STATE VETERINARY COLLEGE, at Cornell University, has issued its opening announcement; at the head of the faculty we find Prof. James Law, F.R.C.V.S., who will occupy the Chair of Principles and Practice of Veterinary Medicine, Veterinary Sanitary Science, and Veterinary Therapeutics; P. Augustine Fish, B.S., B.Sc., D.V.S., as Assistant Professor of Veterinary Physiology, Materia Medica, and Pharmacy; Veranus A. Moore, B.S., M.D., Professor of Veterinary Pathology and Bacteriology, and of Meat Inspection. Among the other members of the faculty and instructors there are no veterinarians; none are very well known to the profession, though all of them have contributed to the high standing of Cornell University in her various departments. Prof. Law, as an instructor and director of such an institution, needs no comment from us, his long years of useful work on behalf of the profession are too well known to all. Prof. Fish has but recently gained his veterinary degree, though he has been well known as an able instructor in other directions. Prof. Moore who now stands at the head of the investigation work of the Bureau of Animal Industry, and who succeeded Prof. Theobald Smith, and who has contributed a great deal to the advanced knowledge of many of the infectious and contagious diseases of the live stock of our country.

The Chair of the Principles of Veterinary Surgery, Zoötechny, Obstetrics, and Jurisprudence is not yet filled, though we are reliably informed that the institution is looking out after one of several very capable men in our country to fill this chair. As with other schools, it starts out with the three-years' course of nine months each, with early decision to advance this in conformity with the other departments of Cornell to four years of nine months each.

The teachings will be of the broadest and most liberal kind, and the new buildings already erected with the facilities they offer, associated with privileges in many of the departments of Cornell, insure a thorough and complete veterinary course. We could have but one additional wish to express in the matter: that she may soon associate with her more veterinarians of the country who are quite well fitted to fill such a position, if sufficient inducements could be offered to them.

As we are partly in press this gratifying news reaches us of the appointment of Prof. W. L. Williams, of Bozeman, Montana, to the vacant chair in the faculty. This is a worthy appointment and the recognition of one who well merits the same. Dr. Williams is peculiarly well equipped and a good all-around man, an earnest student, a thorough worker in all that he does, and one who has won a high place in the esteem of all who know him personally. His writings, well known to the profession at home and abroad, have been such as to command attention and well-deserved comment on all sides. The college strengthens itself by this selection, and the work of its instruction is measurably increased thereby.

